# Sustainable Production Insight Through LCA and LCC Analysis of Injection Overmolded Structural Electronics Manufactured through Roll‐to‐Roll Processes

**DOI:** 10.1002/gch2.202300015

**Published:** 2023-10-25

**Authors:** Minna Räikkönen, Laura Sokka, Lotta Hepo‐oja, Sirpa Nordman, Thomas M. Kraft

**Affiliations:** ^1^ VTT Technical Research Centre of Finland Ltd Visiokatu 4, P.O. Box 1300 Tampere 33101 Finland; ^2^ VTT Technical Research Centre of Finland Ltd Tekniikantie 21, P.O. Box 1000 Espoo 02044 VTT Finland; ^3^ VTT Technical Research Centre of Finland Ltd Kaitoväylä 1, P.O. Box 1100 Oulu 90590 Finland

**Keywords:** LCA, LCC, printed and hybrid electronics, roll‐to‐roll (R2R) manufacturing, sustainability assessment, sustainable production

## Abstract

Printed electronics (PE) have provided new material and application opportunities for devices and systems as well as new manufacturing routes that all need to be considered for commercialization. This paper introduces a case study with universally relevant manufacturing processes and applications in the PE area, focusing on the Life Cycle Assessment (LCA) and Life Cycle Costing (LCC) of the Personal Activity Monitor (PAM) device. In the study, the PAM device's most important costs and environmental impacts during the prototype pilot production and device use phases are identified and assessed. Additionally, the potential environmental impacts of post‐consumption scenarios are considered. The LCA results indicate that the roll‐to‐roll (R2R) assembly of electronics and the R2R injection over‐molding are generally the most prominent production process steps affecting the results. From the LCC perspective, the capitial expenditure (CAPEX) contributor is the R2R assembly pilot line, due to its high investment cost and long operating time compare to other production assets. The traditional electronic components are the major operating expenditures (OPEX), especially the microcontroller units (MCUs) and accelerometers, in contrast to the low impact from the printed electronics. There are several advantages to applying LCA and LCC since they provide explanations of the relationships between cost, environmental, design, and manufacturing characteristics.

## Graphical abstract

This paper introduces a case study in printed electronics, focusing on the Life Cycle Assessment (LCA) and Life Cycle Costing (LCC) of the Personal Activity Monitor device. Results highlight the roll‐to‐roll (R2R) assembly of electronics and the R2R injection over‐molding as key production steps affecting LCA outcomes. The primary cost contributors are the R2R assembly pilot line (CAPEX), MCUs, and accelerometers (OPEX).

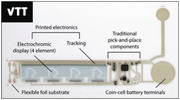

## 1 Introduction

### 1.1 Background

Total global net anthropogenic greenhouse gas (GHG) emissions have continued to grow and the average GHG emissions during 2010–2019 were higher than in any previous decade.^[^ ^1^ ^]^ Climate change is also connected to the loss of biodiversity whose importance has increasingly been recognized in recent years.^[^ ^2^ ^]^ Furthermore, increased awareness and interest in the sustainability of all stakeholders has led to the request for decision support from a sustainability perspective.^[^ ^3^ ^]^ Overall, decision‐makers are faced with the challenge of managing and delivering products and technologies which are not only required to be economically viable but also need to be environmentally sustainable.^[^ ^4^ ^]^ The role of sustainability assessment is especially emphasized in the design phase to inform the developers of the impact of alternative technologies on sustainability objectives and to demonstrate the potential conflicts between sustainability, technology, and business goals.^[^ ^5^, ^6^ ^]^ Assessment of the different sustainability aspects simultaneously provides a deeper understanding of the different angles of sustainability and reduces the risk of shifting problems from one area to another.

Both Life Cycle Assessment (LCA) and Life Cycle Costing (LCC) belong to the group of sustainability assessment tools. First, Life cycle assessment (LCA)^[^ ^7^, ^8^ ^]^ gives a comprehensive picture of the total environmental impacts of a product looking at both direct and indirect impacts. It is an INISO‐standardized method that can be used for assessing the potential environmental impacts of a product or a service throughout its life cycle.^[^ ^9^ ^]^ With LCA, a comprehensive understanding of the environmental impacts of products can be established. For example, it can be applied to recognize which processes or raw materials contribute most to the environmental impacts of a product, and which mitigation actions are most effective in reducing impacts. During the past 10—15 years there has been a lot of development work with the aim of unifying the procedures of LCA conduction and making them more consistent. For example, the Environmental Footprint recommendations published by the European Commission^[^ ^10^ ^]^ refer to LCA‐based general methodology to measure and communicate the potential life cycle environmental impact of a product.^[^ ^11^ ^]^ The methodology contains detailed instructions on how to model and calculate the environmental impacts with methods based on existing, internationally accepted practices, indicators and rules.

Second, life cycle cost projections have an important role in increasing the understanding on economic impacts and cost awareness in the development of novel technologies and implementation strategies.^[^ ^12^, ^13^, ^14^ ^]^ Life cycle costing (LCC)^[^ ^15^, ^16^ ^]^ can be defined as an iterative process of planning, estimating and monitoring all expenses associated with a product, process, sub‐process, or project, including acquisition, operation, and maintenance (Operations & Maintenance, O&M), refurbishment and retirement costs.^[^ ^5^, ^17^ ^]^ A typical case for LCC calculation is a decision‐making situation where the aim is to select an optimal alternative among different options. By considering life cycle costs, decision makers have a better opportunity of optimizing the total cost of ownership and achieving better profitability in the long term. In order to achieve the maximum value for money, all costs incurred over the whole life cycle of a novel technology and related products must be evaluated.^[^ ^18^ ^]^

### 1.2 Sustainability Assessment of Roll‐To‐Roll (R2R) Manufactured Structural Electronics

Printed electronics can provide an inherently more sustainable approach for electronics than the traditional manufacturing technologies. This is mainly due to material types, processing conditions, and material consumption which are widely utilized in industrial and commercial use cases and include numerous domains and applications.^[^ ^19^ ^]^ This is attributed to the significant advances that have occurred over the past three decades for processing and material development for electronics components, systems, and integration techniques. One such domain is hybrid electronics, in which printed electronics are combined with traditional components in a hybrid system (surface mount technology (SMT), on printed backplane), which commonly is on a flexible, or stretchable, substrate.^[^ ^20^ ^]^ To achieve an increased level of integration, and improved material use, structural elements can be made from the hybrid electronic systems which forms a structural electronic element. In this approach, the systems’ functions are seamlessly integrated into a mechanical body resulting in a robust, self‐supporting, integrated product. Structural electronics is particularly relevant when the application emphasizes freedom of design as well as weight and space reduction. This reduction in material use and product weight provides an impact on the sustainability of the structural electronic systems and their eco‐efficiency. The improved sustainability is further compounded by the choice of materials in the printed or coated electronic devices which often use conductive polymers or metal oxides in place of restricted hazardous substances (RoHS).^[^ ^20^ ^]^

Applications for structural electronics have been widely seen in the transportation sector, with many opportunities being explored in health and wellbeing, built environments, and wearables.^[^ ^21^ ^]^ These applications and their technological solutions are based on the combination of printed flexible electronics, and an associated physical integration process that may include thermoforming, injection molding, or lamination in either plastic, glass, paper, concrete, or other material to form the structural element. However, in the development and commercialization of printed and hybrid flexible electronics, most studies have still been more concentrated on the technical or theoretical aspects, and comprehensive sustainability assessments usually have not been on the focus.^[^ ^22^, ^23^ ^]^ There are some studies where the cost perspective is mentioned, but the main focus of these studies has been either on the material, technology or production process development and no cost (LCC) nor investment analysis is conducted.^[^ ^24^, ^25^, ^26^ ^]^ To our knowledge, only very few assessments covering life cycle environmental impacts^[^ ^19^, ^27^, ^28^, ^29^, ^30^, ^31^ ^]^ have been conducted. Overall, it is very important that both LCA and LCC are made, preferably in parallel or even integrated to examine how the environmental sustainability and economic success of products and technologies in the field of printed electronics can be balanced.^[^ ^4^ ^]^

**Figure 1:**
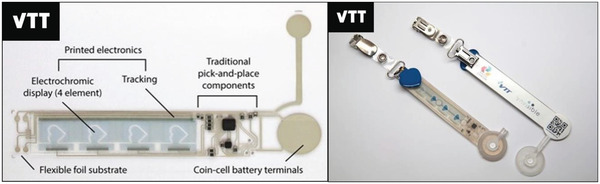
Hybrid electronics device with co‐planar electrochromic display element (left) before injection overmolding and (right) after full assembly.

To perform both the LCA and LCC of a roll‐to‐roll manufactured product, the personal activity monitor (PAM) case study was selected. The Personal Activity Monitor (PAM) device is a compact flexible hybrid electronics unit that provides activity sensing and visual feedback on non‐radiative electrochromic displays. The visual indicator type display consists of 4 hearts that activate in series as the device senses increased movement. The PAM device is injection overmolded with thermoplastic polyurethane (TPU) to provide a seamlessly integrated structural electronics system with removable battery in the form of a badge to be worn on clothes. The PAM platform includes a hybrid integration of traditional components with printed conductive tracks and a co‐planar electrochromic (EC) display fabricated in the roll‐to‐roll (R2R) printing process; it is made using various flexible electronics manufacturing and integration techniques. The electronic backplane and EC display are roll‐to‐roll (R2R) printed. Microcontroller, accelerometer and passive components are pick‐and place assembled. The passive components include capacitors and resistors. The whole device is injection molded to create a structural electronics system^[^ ^2^ ^]^ (**Figure** **1**).

## 2 Research Objective, Methods and Materials

### 2.1 Research Scope, Boundaries and Limitations

**Figure 2:**
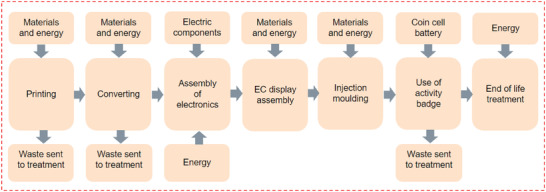
System boundary for the PAM device's life cycle.

The primary objective of the study presented here is to quantify and to evaluate the environmental and economic impacts of implementing advanced electrochromic displays and producing a Personal Activity Monitor (PAM) device. In the study, the most important costs and environmental impacts were identified and assessed, and the main variables of the prototype production process and the PAM device's use phase defined. The use phase in this study involves wearing and utilizing the device to track physical activity and monitor health metrics. Furthermore, for the environmental analysis, the potential impacts of the End‐of‐Life (EoL) scenarios were considered and evaluated (**Figure** **2**). EoL phase refers to the life cycle stage when the device no longer functions properly and thus needs to be replaced. The infrastructure investment cost and activities related to facility construction and transportation were excluded from the LCA and LCC. Additionally, environmental burden from the manufacturing of the production assets were not directly included in the LCA. However, the life cycle inventory (LCI) processes in the ecoinvent database used in the LCA include also the environmental burden related to the capital goods (e.g., construction of powerplants in the case of electricity, or of the factories in the case of the materials).

The research was conducted during the time frame 2021–2022 in close collaboration between researchers in the areas of environmental and economic assessment and printed and hybrid electronics. The underlying research questions were:

- What is the environmental impact and sustainability potential of a PAM device manufactured through roll‐to‐roll processes?
- What are the economic implications and life cycle cost considerations of adopting roll‐to‐roll manufacturing processes for producing PAM devices?

The manufacturing of the device was performed at the Printocent Pilot Factory at VTT Technical Research Centre of Finland Ltd in Oulu, Finland. For this case study used for the LCA and LCC, we extrapolated the device fabrication made on the VTT pilot lines describe in detail in the article by Kololuoma et al.^[^ ^2^ ^]^for R2R scaled up production of nearly 3000 pieces. In that detailed experimental pilot study, the PAM platform was manufactured on the VTT pilot hybrid and structural electronics roll‐to‐roll assembly line for hundreds of pieces. The process steps to manufacture the PAM involve the 1) printing of the backplane on a polyethylene terephthalate (PET) substrate; 2) singulation of the printed circuit board (PCB) on a carrier foil, 3) assembly of surface mounted devices (SMD) electronics, 4) assembly of the electrochromic display element, and then 5) injection overmolding into a urethane elastomer case (Figure 2). The electronic backplane and co‐planar electrochromic (EC) display elements are roll‐to‐roll (R2R) printed on the VTT printing line; with the microcontroller, accelerometer, and passive components (capacitors and resistors) pick‐and place assembled on the R2R SMT assembly line (23 SMD components assembled). Injection overmolding is performed on a R2R fed industrial injection overmolding device designed for roll‐to‐element manufacturing.

The reason for focusing primarily on the device manufacturing in the LCA and LCC is that the production phase typically has significant environmental impacts and cost due to material and resource consumption, energy use, emissions, and waste generation. Additionally, LCA performed already during the development and pilot manufacturing provides valuable information on potential environmental challenges for the product development and the selection of materials and components. From the life cycle cost perspective, this proactive approach prevents costly design changes and optimizations later in the process. Thus, informed decisions aiming to minimize life cycle cost while maximizing the performance and sustainability of the device can be made. In addition to the prototype production, the use of the PAM device was considered in the sustainability assessment. The device has a quite short lifetime, assumed to be two years, and does not require any major maintenance during the usage which reduces significantly the environmental and cost impacts during the use phase. EoL cost was not considered in the LCC as the device is small and thus the disposal cost is relatively small compared to other life cycle cost factors. Furthermore, the disposal of small household electrical and electronic waste can be even free of charge but included in the selling price. However, the details may differ from one country to another. In the LCA, EoL phase considered the treatment processes for generated waste.

### 2.2 Research Methods

#### 2.2.1 Design Science Research Approach

As our research was challenge driven where the scientific methods were applied to find solutions to the practical industrial challenges, the applied research methods are in line with the principles of design science research approach (DSR).^[^ ^32^ ^]^ DSR is particularly suitable for studying an area, which is just emerging, and for creating theoretical and practical understanding of the production process, product and business ecosystem.^[^ ^33^, ^34^ ^]^ In our study, DSR offered for us a possibility to combine data and results from different domains to be incorporated into the case study of the LCA and LCC of the Personal Activity Monitor device. In the context of DSR, case study method^[^ ^35^ ^]^ is often used to investigate the practical application and effectiveness of the developed artifacts in real‐world settings.^[^ ^34^ ^]^ However, it should be considered that our study incorporated one case study of the overmolded structural electronics manufactured through roll‐to‐roll processes. Thus, it may have limited generalizability to other products in the field of printed electronics, manufacturing methods or industries due to its specific focus on roll‐to‐roll processes and injection overmolded structural electronics.

#### 2.2.2 LCA

LCA is a powerful method for understanding the environmental implications of both existing and emerging technologies.^[^ ^28^ ^]^ LCA consists of five different phases: Goal and scope definition; life cycle inventory (LCI), life cycle impact assessment (LCIA), and interpretation.^[^ ^7^ ^]^ The first step on an LCA, goal and scope definition is where the decision is made on what is studied and how. The phase includes also the definition of the functional unit. After goal and scope of the work have been defined, and the functional unit set, the study can proceed to the inventory phase. In the life cycle inventory, data on the inputs and outputs throughout the life cycle of the studied product are collected and calculated.^[^ ^36^ ^]^ Inventory analysis is followed by life‐cycle impact assessment phase where the emissions and resources are grouped to different impact categories (grouping) and converted to common impact units to make them comparable (characterization).^[^ ^37^ ^]^ The conversion into impacts is done by using specific characterization factors and multiplying the inventory results with them. Results of the characterization can then be normalized, which means that the characterized results are divided by a certain reference figure, the global impact caused by one person. The normalized impacts indicate the size of the impacts in comparison to one another, assuming they were all considered to be of equal importance.

In this study, LCA was used to identify and assess the potential environmental impacts of the developed technology and the target consumer devices, including materials development and scaling‐up, prototype displays with innovative substrates, integration of EC with photovoltaics and sensors, printing technology implementation, use and different post‐consumption scenarios (disposal and handling of end products incorporating electrochromics: recycling, reuse, etc). The process life cycles were modelled with the LCA calculation software SULCA.^[^ ^38^ ^]^ In the life cycle impact assessment, impact assessment methods recommended by the Product Environmental Footprint (PEF) were applied.^[^ ^11^ ^]^

#### 2.2.3 LCC

Life Cycle Costing (LCC) is a comprehensive investment appraisal method that assesses the total cost of owning, operating, and maintaining an asset or product over its entire lifetime, helping decision‐makers make financially informed choices. There is an array of standards on life cycle costs available, for example, the standard for general use IEC 60300‐3‐3: 2017: Lifecycle costing for technological systems and ISO 15 663:2021: Petroleum and natural gas industries – Life cycle costing.

The standardized LCC involves several key analysis steps. First, it begins with clearly defining the scope and boundaries of the analysis. This involves identifying all the stages in the life cycle to be included in the analysis and which typically include planning, acquisition, operation, maintenance, and EoL. Establishing these stages is crucial to ensure that all cost elements are appropriately captured. Next, data collection is undertaken to gather all relevant cost information for each stage of the life cycle. This includes obtaining data on initial costs, such as acquisition expenses, and installation costs; O&M costs, encompassing expenses for utilities, labor, consumables, scheduled and corrective maintenance. Finally, End‐of‐Life costs, including disposal, decommissioning, or recycling expenses, are incorporated into the analysis. Monetary values should be given to all costs. Often, for example, engineering and manufacturing estimates for costs are available (market prices). Older estimates available may be updated to the present time of appropriate factors, such as annual inflation factors. Comparison of cash flows from different periods can be achieved by incorporating the time value of money. After completing the assessment, the results are ready to be put to practice. The results of LCC can be characterized by key performance indicators (KPIs); e.g., total life cycle costs are the sum of discounted costs for the calculation period (lifetime). Furthermore, if calculation parameters are highly uncertain, sensitivity analysis can be performed, e.g., by Monte Carlo simulation which is useful especially when the uncertainty of several calculation parameters needs to be considered at the same time.

Within our study, the LCC (life cycle cost) analytic definition is:

(1) $$LCC=C_{C}+C_{M}+C_{AM}+C_{EC}+C_{EL}+C_{L}+C_{U}\left(€\right)$$

The economic parameters and the input data considered within this definition are the initial cost of the machinery (C_C_), the cost of materials (C_M_), the cost of auxiliary materials (C_AM_), the cost of electronic components (C_EC_), the cost of electricity consumption (C_EL_), the cost of labor (C_L_), and the cost of use of the PAM device (C_U_).

In addition, the comparison of cash flows from different periods should be and is included in the study. The purpose of discounted cash flow analysis is to determine the net present value (NPV) of different future cost flow streams. Discounting formula primarily converts the future cash flows to present value by using the discounting factor.

(2) $$P_{V}=\;P_{S}/{\left(1+R\right)}^{n}$$

where: P_V_ is the value at the end of n years; P_S_ is the value at start of investment; R is the interest rate; n is the number of years of investment.

### 2.3 Materials

Both LCA and LCC modelling were conducted for 2930 PAM devices which equals to PET area of 30 m^2^ with the repeat length of app. 41 cm and web width 30 cm. While the device is during this study produced in a pilot laboratory, assumptions in the modelling were made regarding number of devices produced per 1 m^2^, and on the amount of energy used for the production. Data used in the LCA was mainly based on the ecoinvent database (Table S1, Supporting Information, Wernet et al., 2016). The process and cost data used in the LCC were gathered and communicated by the experts in the field of printed and hybrid electronics and completed by using the literature references and price catalogues (Table S1, Supporting Information). In addition to the variables presented in Table S1 (Supporting Information), the following parameters were applied in the LCC:

*Work schedule*: In the study, the work schedule of 7,5 h and 5 days in a week was considered. For the upscaling case, to be able to maximize the production volume, the continuous service 24/7 was assumed.
*The discount rate (%)*: Different discount rates apply to different companies and to different decision‐makers and to different situations. In this study, 8% was applied.
*Life cycles*: The estimates of printing screen life cycle for Ag paste is 1500 m and for poly(3,4‐ethylenedioxythio‐phene) polystyrene sulfonate (PEDOT:PSS) paste 500 m which were also taken into account in the analysis. A coin‐cell battery CR2032 220 mA was considered and assumed to last one year if the PAM device is used 12 h a day. The lifetime for the device is the same as used in the LCA, 2 years.

The production assets that were included in the LCC were R2R‐printer, R2R‐converter, R2R‐assembly and R2R injection molding machine (**Table** **1**). The service life of each machine was assumed to be 20 years which relates to 41 600 h. The machine operating times for 2930 devices varied from 3,5 h (R2R‐converter) to over 100 h (R2R‐assembly). The assembly of EC display was done manually and thus excluded from Table 1. It should be noted that the pilot line was used, and all the pilot lines are unique, like the Printocent Pilot Factory in this study. The pilot line in question is optimal for flexible manufacturing of small batches, and thus the machine times and overall process times in Table 1 are not optimized for a dedicated product.

**Table 1:** Production assets in the pilot factory.

Capital assets	Investment cost [€]^2^ ^a)^	Operating hours [h] for 2930 devices	References
R2R‐printer R2R‐converter R2R‐assembly R2R injection molding machine	410 000 620 000 1 100 000 290 000	20 3,5 105 22,5	Prices are from the procurement system, supplemented by expert judgment. Operating hours are based on the experimental work in the pilot factory.

^a)^ Investment costs are approximate values within the order of magnitude of the specialized unique pilot line equipment.

## 3 Sustainability Assessment of the PAM Device

### 3.1 LCA and LCC Calculations

LCA results were calculated for different production phases, and for different categories of materials used. LCC considered three scenarios: 1. one PAM device, 2. 2930 devices and 3. hypothetical upscaling study over a period of 5 years for an assumed yearly production of 240 000 devices which relates to the maximum production capacity of the current machines in the pilot factory.

Furthermore, an additional scenario was calculated both in the LCA and LCC in order to analyze how the environmental and life‐cycle cost impacts would change if indium tin oxide (ITO)‐PET was used as a substrate instead of PET substrate. ITO was chosen here to improve the conductivity of the transparent EC electrode which is typically used in this kind of device. Electrochromic displays based on the conductive polymer PEDOT:PSS can be fabricated with, or without, a transparent conductive electrode (such as indium tin oxide, ITO). This is due to the electronic nature of the PEDOT electrochromic polymer film. Switching times can be increased with the ITO, however, with the increased material costs and processing steps it is not necessary. In addition to ITO, the scenario included etching paste and electricity needed for the process (Table S2, Supporting Information).

### 3.2 LCA Results

**Figure 3:**
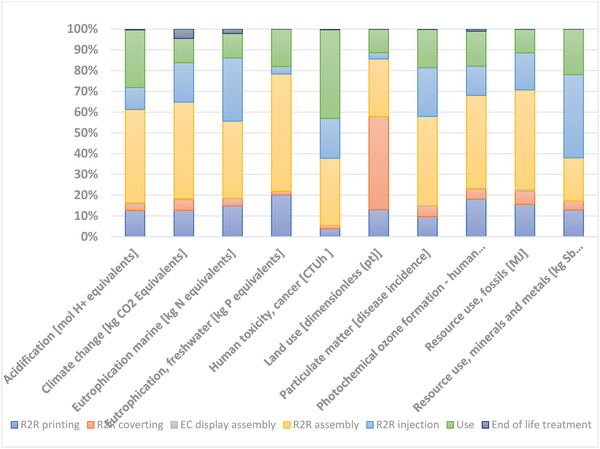
Life cycle impact assessment results for the PAM device. Division by production (production steps), use and EoL life cycle stages.

**Figure 4:**
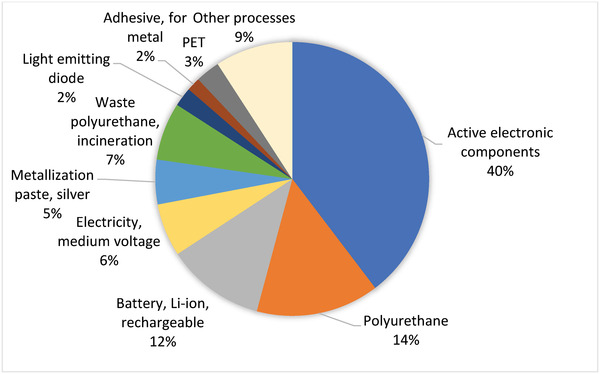
Main processes contributing to climate impacts.

In the following, LCA results are presented divided by different life cycle stages and by different types of resources (**Figure** **3** and **4**). Life cycle stage is a term used in LCA, and it refers to the different parts of a product's life cycle. Stages can be defined differently, depending on the product analyzed, and the study's aims. In the present case, five of the stages refer to the different production steps, followed by use and end‐of‐life stages. The results for different life cycle stages show that the R2R assembly of electronics and the R2R injection molding are generally the main production steps affecting the results, with the components needed for the R2R assembly having the largest contribution in most impact categories. This mainly stems from the production of electronic components (both active and passive components), and the SMD resistor needed for the hybrid system. This is also visible in **Figure** **4**, where electric components are a large contributor to the total impacts. The high contribution of R2R conversion to land use impacts results from the paper which is assumed to be the raw material of the Pressure Sensitive Adhesive (PSA) liners.

Furthermore, the use phase is also a fairly large contributor to the results, causing 10–40% of the impacts in most impact categories with acidification and human toxicity having the largest impacts (Figure 3). These impacts primarily originate from the battery production.

The processes contributing to the climate impacts (carbon footprint) of the device were studied in more detail (Figure 4). For the climate impacts, the active electronic components were the main contributors, causing 40% of the total impacts; in contrast to the low‐impact organic materials and printed Ag. The active components are the microcontroller and accelerometer which were modelled using global average data from the ecoinvent database. The significant impact of active components can be explained by the less environmentally sound semi‐conductor industry materials and processes used in traditional electronics production, as compared to the more benign materials and R2R manufacturing used in the PAM use case. The electronic components contain critical metals and other elements whose mining and production is typically very energy intensive. Also, polyurethane and Lithium‐ion (Li‐ion) battery production caused 14 and 12 percent of the emissions, respectively.

**Figure 5:**
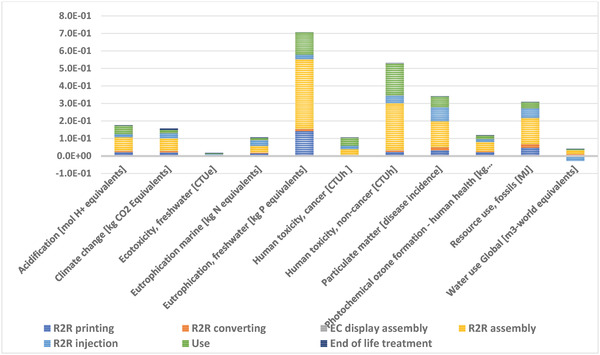
Normalized impacts divided to the different life cycle stages.

As explained in Chapter 2.2.2, normalization in LCA means that the characterized results are divided by a certain reference figure, in this case the global impact of caused by one person. The normalized impacts indicate the size of the impacts in comparison to one another, assuming they were all considered to be of equal importance. Of the normalized impacts, use of resources, both fossil fuels and minerals, eutrophication of freshwaters and human toxicity are the largest (**Figure** **5**). However, it should be noted that that all the impacts are typically not considered equally important by society but some of them are considered to be more urgent, such as climate change.

**Figure 6:**
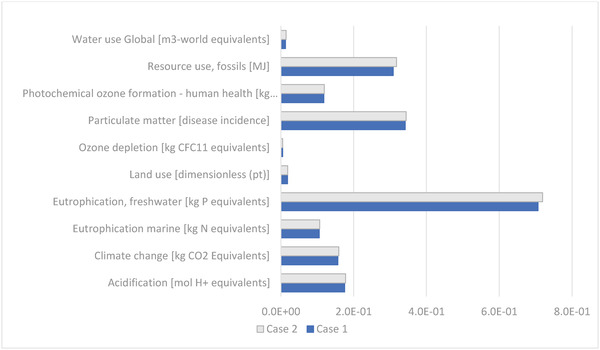
Comparison of normalized impacts in the base case (Case 1) and another case (Case 2) where ITO (indium tin oxide) ‐PET is used as a substrate instead of PET.

The impacts of adding transparent conductive electrode in the form of ITO into the EC display are shown in **Figure** **6** where the two cases are compared. Even though the production emissions of ITO are quite high, c. 15 kg CO_2_/kg ITO in comparison to c. Three kg CO_2_/kg PET, the amount of ITO needed is so small that the impacts of it and the additional etching paste increases the environmental impacts only slightly.

### 3.3 LCC Results

In order to approve the target device from the cost perspective, the total life cycle, discounted life cycle cost as well as annual cost and discounted cumulative cost were calculated as result indicators of the LCC. Key financials combined with the results of the PAM device are presented in the following table and figures.

In **Table** **2**, capital expenditures (CAPEX) of production assets required in the manufacturing was considered and allocated for the PAM devices by using the operating time of a machine for producing a certain number of devices. It should be taken into consideration that the pilot line set‐up was used and thus the production assets in the study are not industrial‐scale machines. In addition, the assembly of EC display was done manually and thus not considered in CAPEX. Typically, the machine capacities are shared between the different products produced which needs to be considered when allocating the CAPEX to production assets. In the industrial scale production, the production assets with the longest operating time might form bottlenecks that reduce the capacity of the whole chain. Therefore, additional machine investments might be needed.

**Table 2:** Key result indicators and the LCC results.

OPEX	Cost [€] / one PAM device	Cost [€] / 2930 PAM devices	Yearly production 240 000 devices, calculation period 5 years, discount rate 8%	
			Not discounted	Discounted
OPEX – Materials (€)	1,11	3 000	1 300 000	1 000 000
OPEX – Auxiliary materials (€)	0,08	200	95 000	75 000
OPEX – Electronic components (€)	2,20	6 500	2 500 000	2 000 000
OPEX – Electricity consumption (€)	0016	50	20 000	15 000
OPEX – Labour (€)	1,38	4 000	1 650 000	1 300 000
OPEX – Use phase (€)	1,32	4 000	1 600 000	1 250 000
OPEX TOTAL	6,10	17 750	7 165 000	5 640 000

CAPEX	Cost [€] / one PAM device	Cost [€] / 2930 PAM devices	Yearly production 240 000 devices, calculation period 5 years, discount rate 8%	
			Not discounted	Discounted
CAPEX total (€)	2 400 000 €	2 400 000 €	2 400 000	2 400 000
CAPEX allocated based on the machine operating hours during calculation period (5 years)	1,0	3 000	1 250 000	1 250 000

As evidenced in the Table 2, operational expenditures (OPEX) in the study comprised the cost of materials and components, energy, labor related to manufacturing of the device, cost use associated with the use of the device, and other operating cost. Raw materials were divided into materials, auxiliary materials, and electronic components. The material cost for various production amounts were calculated based on the process and cost data on the production process in the pilot factory. The energy cost was calculated based on the electricity consumption of the production assets and the electricity price. The direct cost of labor included the cost of wages and benefits for employees who are directly involved in the production. The working hours allocated are linked to the operating time of the machines. Electronic components are typically bought in reels, which reduces the unit prices. This was also an assumption in this study.

**Figure 7:**
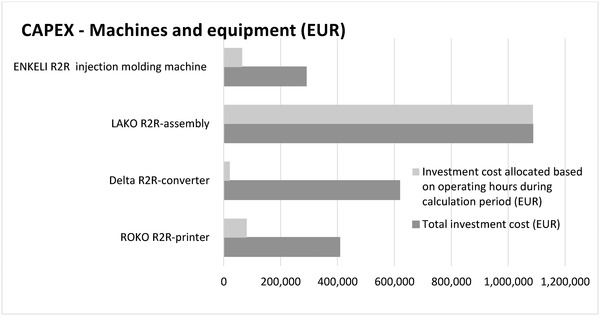
CAPEX – both total and allocated based on the machine operating hours. Calculation period 5 years.

**Figure 8:**
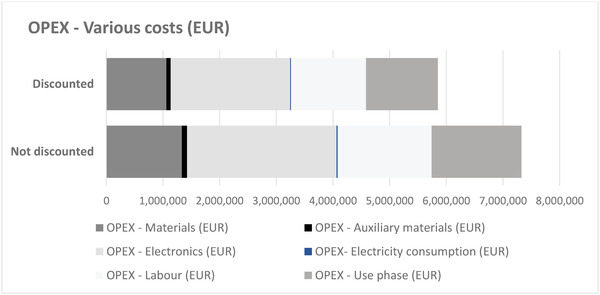
OPEX total – yearly production 240 000 devices, calculation period 5 years, discount rate 8%.

Based on the LCC results, the most significant production assets, materials, and components in terms of CAPEX and OPEX are:

- CAPEX – Machines and equipment: R2R‐assembly, R2R‐converter, R2R‐printer (see **Figure** **7**)
- OPEX (see **Figure** **8**)
  ○ Materials: PSA, TPU, EC display: EC‐ink, non‐conductive adhesive
  ○ Auxiliary materials: Printing screens, water, die cut tool
  ○ Electronic components: MCU, accelerometer, light emitting diode (LED) element
  ○ Electricity consumption: Electricity consumption of R2R‐printer, R2R‐assembly
  ○ Labor: Operator: R2R assembly, R2R injection molding machine, and R2R printer
  ○ Use phase: Coin cell battery

The identification and valuation of the CAPEX and OPEX include a varying amount of uncertainty and the expected costs are inherently uncertain. For example, most O&M costs can vary a lot when scaling up from the pilot scale to large‐scale industrial production.

However, as a typical LCC result indicator is a point estimate, e.g., a numerical value, it does not take into account the uncertainty. If the expected variation of the LCC is large, as in the case of novel technologies and products, like in the case of printed electronics, the sensitivity analysis will be a very useful tool to demonstrate the impact of changes.

The example of the sensitivity analysis in our study was to examine the impact of using the ITO‐PET substrate and etching paste instead of PET substrate. In addition to the cost increase in the used feedstock, this change has an impact on the R2R printing line's energy consumption, amount of printing screens and water consumption in the R2R printing step, and labor cost of R2R printer which all also increase the cost. However, the most cost increase arises from ITO‐PET substrate and etching paste. Based on the sensitivity analysis results, for 5 years timeframe and yearly production of 240 000 devices, the difference in total LCC between the original and sensitivity analysis is that the option where PET‐ITO substrate and etching paste is used the cost increase is app. 440 000 € (not discounted)/350 000 € (discounted) compared to the option using PET substrate.

## 4 Discussion

The life cycle assessment results for the PAM device indicate that the R2R assembly of electronics and the R2R injection molding are generally the main life cycle phases affecting the results, with the components needed for the R2R assembly having the largest contribution in most impact categories. R2R printing and converting, and the EC display assembly have smaller impacts. The use phase is also a fairly large contributor to the results, causing 10–40% of the impacts in most impact categories with the human toxicity impacts being the most important ones. These impacts mainly originate from battery production. From the LCC perspective, the CAPEX contributor is the R2R‐assembly, due to its high investment cost and long operating time compared to the other production assets in the manufacturing of the PAM devices. In the case study, the assembly of EC display was done manually. If the EC display assembly will be automated in future, the asset investment should be accounted for in the LCC accordingly. Regarding the OPEX, the main expense arises from electronic components, especially from MCUs and accelerometers. The second OPEX contributor is the labor cost, followed by the cost related to coin cell battery in the use phase and materials, especially PSA, TPU, and EC ink. Electricity consumption of the production assets and auxiliary materials do not contribute significantly to the OPEX.

As the calculations are done based on data for lab scale pilot production of devices, it should be considered that if large scale production was studied in a more detail, it would probably have an impact on the LCA and LCC results. The increase in production will induce, e.g., changes in material and electricity consumption and efficiency, and bring cost advantages, both from CAPEX and OPEX perspectives. In the present case, the losses of materials in production are quite high, even more than double the amount used for the device in some cases. Such practice would most likely not occur in industrial scale production. Furthermore, the energy use of the machines is not optimized for this PAM device. Thus, the energy consumption per device would probably be lower on an industrial scale production. Yet, even at this scale of production, the analysis gives several important results, e.g., on the factors mainly affecting the impacts, which again provides important information for product development.

There are many advantages in applying both LCA and LCC as they provide explanations of the relationships between cost, environmental, design and production parameters. Thus, they can also contribute to possible design and manufacturing changes. For example, life cycle costing provides valuable information on possibilities to minimize the cost and to improve the competitiveness by increasing the added value of a conventional product where printed electronics elements are integrated. It plays especially a role in cost reduction by identifying high‐cost contributors and by supporting the creation of a common understanding of the cost impacts. It is also very valuable to analyze the environmental impacts already at the design phase in order to be able to respond to them in the product design by directing the material and energy use of the product to options with less environmental impacts.

However, it should be noted that it is not straightforward to analyze the life‐cycle costs and environmental impact of hybrid and structural electronic systems and devices. The first challenge to overcome is to establish a structure that takes into account all relevant factors related to the system and a product where typically several companies are involved in the production. Another challenge is the availability and reliability of data for calculations and analyses. The data related issues are particularly highlighted if the LCC and LCA are done in the initial phase of development and in the pilot factory environment where the uncertainty is high. This was also the case in this study. In addition, there is still very little data from real deployments of hybrid and structural electronics, and existing data points typically originate from pilot factories and demonstration projects.

In practice, it is also critical to understand the major assumptions behind the evaluations and to have a comprehensive understanding on how the assessments are carried out and what the analysis outcomes really mean. Sensitivity analysis may be utilized to evaluate the robustness of the LCC and LCA outcomes to changes in various factors. The sensitivity analysis was also part of this research study and revealed that the most cost increase was caused by the PET‐ITO substrate and etching paste when compared to the case where the PET substrate is used. For the environmental impacts the changes resulting from the use of ITO were very small, and also the cost increase caused PET‐ITO and etching paste was small compared to the total OPEX.

Despite the significant progress, the printed electronics has not yet realized commercial array scale deployment. However, the markets for products to interact with the environment in new ways is growing quickly. This could lead also to significant learning with experience. Moreover, making good use of the advantages and value‐added of current developments will allow to ensure competitiveness in the commercial market. And by further examining the possibilities to replace materials and components that are most expensive or have the highest harmful environmental impacts, and to increase the production process performance, significant reductions in cost or environmental impacts are achievable.

## 5 Conclusion

This paper has presented the LCA and LCC analysis of implementing advanced electrochromic displays and producing a personal activity meter device. In the study, the most important costs and environmental impacts were identified and assessed, and the main variables in each phase of the production process and use phase of the PAM device life cycle were defined. The study was conducted at “Printocent Pilot Factory” at VTT.

In developing and commercializing of printed and hybrid flexible electronics, most studies have yet concentrated on the technical or theoretical aspects. However, it is very important that both LCA and LCC also conducted already in the early design and development phase. There are many advantages to applying LCA and LCC as they provide explanations of the relationships between cost, environmental, design, and production parameters. Thus, they can also contribute to possible design and manufacturing changes. Overall, future research can examine how LCA and LCC may be further developed and utilized as a foundation for future methodological breakthroughs in sustainability assessment in the field of printed and hybrid flexible electronics.

## Conflict of Interest

The authors declare no conflict of interest.

## Supporting information

Supporting Information

## Data Availability

The data that support the findings of this study are available in the supplementary material of this article.
