# Supplementary material for: Sustainable Production Insight Through LCA and LCC Analysis of Injection Overmolded Structural Electronics Manufactured through Roll‐to‐Roll Processes
Source: Glob Chall. 2023 Oct 25;7(11):2300015. doi: 10.1002/gch2.202300015 (PMC10632665; doi:10.1002/gch2.202300015)
Supplement: Supplementary file 1 — Supporting Information [file GCH2-7-2300015-s001.pdf]

Global Challenges
Open Access

Supporting Information

for *Global Challenges.*, DOI 10.1002/gch2.202300015

Sustainable Production Insight Through LCA and LCC Analysis of Injection Overmolded Structural Electronics Manufactured through Roll-to-Roll Processes

Minna Räikkönen, Laura Sokka, Lotta Hepo-oja, Sirpa Nordman and Thomas M. Kraft*

Supporting Information

Table S1. Materials, energy, and prices used in the sustainability assessment.

Inputs	For one PAM device	Unit	Assumptions used in modelling	Ecoinvent process used for LCI data
**PET substrate**	102	cm^2	density 0.01832 g/cm^2<br>Price: 3.5 €/m^2	market for polyethylene terephthalate, granulate, amorphous [GLO]; market for extrusion of plastic sheets and thermoforming, inline [GLO]
**Printing screens**	0.00009	pcs	stainless steel, lifetime 500m for PEDOT:PSS paste, lifetime 1500m for Ag paste<br>Price: 350 €/pcs	market for steel, chromium steel 18/8, hot rolled [GLO]; market for impact extrusion of steel, cold, 1 strokes [GLO]
**Ag paste**	0.06	g	density 2.0 g/cm^3<br>Price: 1.6 €/g	market for metallization paste [RER]
**PEDOT:PSS paste**	0.08	g	density 1.0032 g/cm^3<br>Modelled based on [40]<br>Price: 0.42 €/g	market for bromine [GLO]; market for carbon disulphide [GLO]; market for 1-butanol [GLO]; market for polystyrene, general purpose [GLO]; market group for electricity, medium voltage [RER]; market group for tap water [RER]
**Washing agents**	1.0	ml	Assumed 50/50 composition between non-ionic surfactant production, ethylene oxide derivate and non-ionic surfactant production, fatty acid derivate<br>Price: 10 €/ l	non-ionic surfactant production, fatty acid derivate [GLO]; non-ionic surfactant production, ethylene oxide derivate [GLO]
**PET carrier (to be removed)**	102	cm^2	density 0.01832 g/cm^2<br>Price: 3.5 €/m2	market for polyethylene terephthalate, granulate, amorphous [GLO]; market for extrusion of plastic sheets and thermoforming, inline [GLO]
**PSA (2-sided adhesive film)**	47.89/	cm^2	weight 0.1756 g/unit<br>50% adhesive 50% PET<br>Price: 28 €/m^2	market for adhesive, for metal [RER]; market for polyethylene terephthalate, granulate, amorphous [GLO]; market for extrusion of plastic sheets and thermoforming, inline [GLO]
**PSA liner x 2 (to be removed)**	85.21	cm^2	weight 0.1244 g/unit	market for paper, woodfree, uncoated [RER]
**Die cut tool**	0.000034	pc	Lifetime 1000m<br>Price: 350 €/ pcs	excluded from the study
**EC-ink (electrolyte)**	0.3	ml	Modelled as PEDOT:PSS based García-Valverde et al. 2010<br>Price: 500 €/kg	market for bromine [GLO]; market for carbon disulphide [GLO]; market for 1-butanol [GLO]; market for polystyrene, general purpose [GLO]; market group for electricity, medium voltage [RER]; market group for tap water [RER]
**PET-cover**	12.77	cm^2	density 0.01832 g/cm^2<br>Price: 3.5 €/m2	market for polyethylene terephthalate, granulate, amorphous [GLO]; market for extrusion of plastic sheets and thermoforming, inline [GLO]
**LED**	1.0	pcs	weight 38.0 mg/pcs<br>Price: 2 858 € /reel, 30 000 pcs/reel	market for light emitting diode [GLO]

**Microcontroller**	1.0	pcs	weight 60.8 mg/pcs<br>Price: 6 628 € /reel, 6 000 pcs/reel	market for electronic component, active, unspecified [GLO]
**Accelerometer**	1.0	pcs	weight 78.0 mg/pcs<br>Price: 7 974 € /reel, 8 000 pcs/reel	market for electronic component, active, unspecified [GLO]
**Capacitor**	3.0	pcs	weight 6.3 mg/pcs<br>Price: 114 € /reel, 100 000 pcs/reel	market for capacitor, for surface-mounting [GLO]
**Resistor**	12.0	pcs	weight 1.5 mg/pcs<br>Price: 7.5 € /reel, 50 000 pcs/reel	market for resistor, surface-mounted [GLO]
**Isotropic conductive adhesive**	0.05573	ml	Price: 100 €/cm3	market for adhesive, for metal [RER]
**Non-conductive adhesive**	0.1932	ml	Price: 2.4 €/g	market for adhesive, for metal [RER]
**IPA washing agent**	0.5	ml	Assumed 50/50 composition between non-ionic surfactant production, ethylene oxide derivate and non-ionic surfactant production, fatty acid derivate<br>Price: 9 €/l	non-ionic surfactant production, fatty acid derivate [GLO]; non-ionic surfactant production, ethylene oxide derivate [GLO]
**TPU**	12.0	g	Price: 20.1 €/kg	market for polyurethane, rigid foam [RER]
**Water**	0.409	l	density 1.0 g/cm3<br>Price; 4 €/m3	market group for tap water [RER]
**Electricity**	0.087	kWh	European mix<br>Price: 0.18 € / kWh	market group for electricity, medium voltage [RER]
**Battery**	3	g	Small coin cell battery with lifetime of 1 year<br>Price: 0.66 €	market for battery, Li-ion, rechargeable, prismatic [GLO]
**Operator salary**	-	-	-	-

Waste output	For one PAM device	Unit	Assumptions used in modelling	Ecoinvent process used for LCI data
**Waste PET substrate**	204.2	cm2	From ROKO R2R printing and Delta R2R converting	treatment of waste polyethylene terephthalate, municipal incineration [CH]
**Waste printing screens**	0.00009	pcs	From ROKO R2R printing	treatment of hazardous waste, hazardous waste incineration [CH]
**Waste washing agents**	1	l	From ROKO R2R printing	treatment of wastewater, average, capacity 1E9l/year [Europe without Switzerland]
**Waste PSA**	43.75	cm2	From Delta R2R converting	treatment of waste polyethylene terephthalate, municipal incineration [CH]
**Waste PSA liner**	85.21	cm2	From Delta R2R converting	treatment of waste polyethylene terephthalate, municipal incineration [CH]; treatment of waste paperboard, municipal incineration [CH]
**Waste isotrophic adhesive**	0.05417	ml	From LAKO R2R assembly	treatment of hazardous waste, hazardous waste incineration [CH]

**Waste non-conductive adhesive**	0.1392	ml	From LAKO R2R assembly	treatment of hazardous waste, hazardous waste incineration [CH]
**Waste PAM device at EOL, PET part**	4.807	g	50% is assumed incinerated 50% is assumed landfilled	treatment of waste polyethylene terephthalate, municipal incineration [CH]; treatment of waste polyethylene terephthalate, sanitary landfill [CH]
**Waste TPU**	6.5	g	From R2R injection, all assumed to be incinerated	treatment of waste polyurethan, municipal incineration [CH]
**Waste PAM device at EOL, electronic part**	4.252	g	50% is assumed incinerated 50% is assumed landfilled	treatment of waste electric and electronic equipment, shredding [GLO]
**Waste TPU, EOL**	5.5	g	100% assumed to be incinerated	treatment of waste polyurethane, municipal incineration [CH]
**Wastewater**	4.91	l	From R2R injection	treatment of wastewater, average, capacity 1E9l/year [Europe]

Table S2. Materials and energy needed for use of PET-ITO.

Inputs	For one PAM device	Unit	Assumptions used in modelling	Ecoinvent process
**PET substrate**	102	cm^2	density 0.01832 g/cm3<br>PET-ITO price: 30 €/m2	market for polyethylene terephthalate, granulate, amorphous [GLO]; market for extrusion of plastic sheets and thermoforming, inline [GLO]
**Indium tin oxide (ITO)**	2.61E-09	g	7,14 g/cm3	indium tin oxide powder production, nanoscale, for sputtering target [RER]
**Etching paste**	0.26	g	Structure of etching paste estimated based on the Safety Data Sheet of etching paste produced by Columbus Chemical Industries. Main components oxalic acid and water. Data on these taken from the ecoinvent database.<br>Price: 352 €/kg	Market for oxalic acid [GLO]; market group for tap water [RER]
**Printing screens**	0.00009	pcs	stainless steel, lifetime 500m for PEDOT:PSS	market for steel, chromium steel 18/8, hot rolled [GLO]; market for impact extrusion of steel, cold, 1 strokes [GLO]

			paste, lifetime 1500m for Ag paste<br>Price: 350 €/pcs	
**Waste PET-ITO**	78.25	cm2		treatment of waste polyethylene terephthalate, municipal incineration [CH]
